# The role of Immune cells in Alzheimer's disease: a bidirectional Mendelian randomization study

**DOI:** 10.3389/fnagi.2024.1433691

**Published:** 2024-07-15

**Authors:** Erdong Zhang, Tingting Chen, Yanqin Chen, Chenxiang Long, Ling Tao, Xiangchun Shen, Fengqiu Dai

**Affiliations:** ^1^The Key Laboratory of Optimal Utilization of Natural Medicine Resources, School of Pharmaceutical Sciences, Guizhou Medical University, Guiyang, Guizhou, China; ^2^The State Key Laboratory of Functions and Applications of Medicinal Plants, Guizhou Medical University, Guiyang, Guizhou, China; ^3^The High Efficacy Application of Natural Medicinal Resources Engineering Center of Guizhou Province, School of Pharmaceutical Sciences, Guizhou Medical University, Guiyang, Guizhou, China; ^4^The Pharmacy Department, Guiyang Maternal and Child Health-Care Hospital, Guiyang, Guizhou, China; ^5^Department of Anatomy, School of Basic Medical Sciences, Guizhou Medical University, Guiyang, China

**Keywords:** Alzheimer's disease, immune cells, Mendelian randomization study, HLA DR on B cells, CD4^+^CD8 dim T cells, CD14^+^CD16^−^ monocyte

## Abstract

**Background:**

Alzheimer's disease (AD) is a leading cause of dementia, characterized by the accumulation of amyloid-beta (Aβ) and hyperphosphorylated tau proteins, leading to neuroinflammation and neuronal damage. The role of the immune system in AD pathogenesis is increasingly recognized, prompting an exploration of the causal relationship between immune cells and AD by using Mendelian randomization (MR) approaches.

**Methods:**

Utilizing genome-wide association study (GWAS) data from European cohorts, we conducted an MR study to investigate the causal links between immune cell phenotypes and AD. We selected single nucleotide polymorphisms (SNPs) associated with immune cell traits at a genome-wide significance threshold and applied various MR methods, including MR Egger, Weighted median, and inverse variance weighted analysis, to assess the causality between 731 immune phenotypes and AD.

**Results:**

Our MR analysis identified 15 immune cell types with significant causal relationships to AD pathogenesis. Notably, the absolute count of CD28^−^CD4^−^CD8^−^ T cells and the expression of HLA DR on B cells were linked to a protective effect against AD, while 13 other immune phenotypes were identified as contributing to the risk factors for the disease. The causal effects of AD on immunophenotypic traits are predominantly negative, implying that AD may impair the functionality of immune cells. Validation through independent datasets, such as FinnGen and GCST90027158, confirmed the causal association between six specific immune cells and AD.

**Conclusion:**

This comprehensive MR study elucidates the intricate network of causal relationships between diverse immunophenotypic traits and AD, providing novel insights into the immunopathogenesis of AD. The findings suggest potential immunological targets that could be leveraged for early diagnosis, disease monitoring, and therapeutic intervention.

## 1 Introduction

Alzheimer's Disease (AD) is a major cause of dementia and is swiftly emerging as one of the most financially draining, lethal, and burdensome health issues of our time (Livingston et al., 2020). With the escalating trend of an aging global population, the incidence and prevalence of AD are on a continuous upswing, presenting unprecedented challenges for individuals, families, and society at large (Alzheimer's disease facts and figures, 2024). Data from the Alzheimer's Association and the World Health Organization (WHO) indicate that around 55 million individuals worldwide are affected by dementia, with projections estimating a doubling of this number by 2050 (Alzheimer's disease facts and figures, 2024). Within this demographic, AD, the most prevalent neurodegenerative condition, is responsible for approximately 50% to 70% of all neurodegenerative dementia cases (2024). The pathophysiology of AD is marked by the abnormal accumulation of two key proteins: amyloid-beta (Aβ) and tau. These aberrant protein deposits manifest as amyloid plaques and neurofibrillary tangles (NFTs) within the brain, culminating in impaired neuronal function and cell death (Villemagne et al., 2013). The presence of Aβ plaques and NFTs prompts the activation of brain immune cells, notably microglia, initiating a cascade of chronic neuroinflammation that further aggravates neuronal damage (Rajmohan and Reddy, 2017). Consequently, therapeutic strategies for AD are centered on reducing Aβ plaque accumulation, preventing the abnormal phosphorylation of tau protein, mitigating neuroinflammation, and enhancing neuronal function. The ultimate goal of these interventions is to halt or reverse the disease's pathological trajectory, thereby aiming to enhance cognitive abilities and the overall quality of life for patients.

The etiology of AD is exceptionally intricate, encompassing a spectrum of factors such as genetics, environmental exposures, and lifestyle choices. The role of the immune system in this context has introduced novel dimensions to our understanding of the condition (Frost et al., 2019). The activation patterns of the immune system within AD potentially provide pivotal insights for the early detection of the disease (Liddelow et al., 2017). Research has indicated the presence of a chronic inflammatory process within the brains of individuals with AD, a process that may initiate prior to the observable decline in cognitive abilities (Xiong et al., 2021). Specifically, microglia—the principal immune cells of the central nervous system—are intimately involved in the genesis and removal of Aβ (Sun et al., 2023b). The sustained activation of microglia in the brain tissue of AD patients can lead to the release of inflammatory mediators that intensify neuroinflammation and may inflict damage upon neurons (Xiong et al., 2021). Consequently, the surveillance of microglial activation levels could be instrumental in the preemptive identification of individuals at elevated risk for AD, prior to the onset of clinical symptoms. Furthermore, the heterogeneity and complexity inherent to immune cells present unique challenges for the therapeutic management of AD (Keren-Shaul et al., 2017). Diverse immune cell types may exert distinct effects within the context of AD, and even within a single cell type, varying activation states can lead to markedly different outcomes (Feng et al., 2021; Chen and Holtzman, 2022; Jorfi et al., 2023b). Therefore, the monitoring and regulation of immune cell activation could serve as a critical instrument in disease surveillance, facilitating the evaluation of disease activity and the rate of progression.

Traditional research has encountered significant challenges in delineating a causal link between immune cells and AD, largely attributable to constraints in study design and the confounding effects of factors such as age, gender, genetic lineage, lifestyle elements, and the presence of comorbidities, all of which can influence immune cell function and the risk profile for AD (Nebel et al., 2018; Xia et al., 2018; Zhang et al., 2021). While observational study methodologies, including cross-sectional and cohort studies, have furnished critical epidemiological understanding of AD, the alteration of immune cells could be both an effect and a precipitant of the disease, obfuscating the identification of a definitive causal sequence (Yu et al., 2020; Xu and Jia, 2021; Rajesh and Kanneganti, 2022). Mendelian Randomization (MR) studies, which utilize genetic variants as instrumental variables to investigate causality, present a methodological solution (Chen et al., 2022). This MR approach uses genetic variants, which are stochastically allocated during gametogenesis, mirroring the random assignment characteristic of randomized controlled trials, thereby diminishing the confounding effects (Emdin et al., 2017; Larsson et al., 2023). The inherent fixity of genetic variants, coupled with their extraneousness to disease status, positions MR as a robust tool for minimizing the risk of reverse causal inference (Chen et al., 2022). MR studies can capitalize on data from extensive Genome-Wide Association Studies (GWAS), offering genetic variants intimately linked to discrete traits and amplifying the statistical vigor of the research (Emdin et al., 2017; Larsson et al., 2023). Utilizing MR analysis, we have probed into the causal nexus between immune cells and the genesis and trajectory of AD, establishing a causal relationship between six distinct immune cell types and the evolution of AD. These findings not only illuminate potential therapeutic targets for AD but also herald the potential for personalized medical approaches. By scrutinizing patients' immunological profiles, including the phenotype and functionality of immune cells, it becomes feasible to anticipate individual responses to targeted treatments, enabling a bespoke therapeutic strategy.

## 2 Materials and methods

### 2.1 Data approval

**Figure 1:**
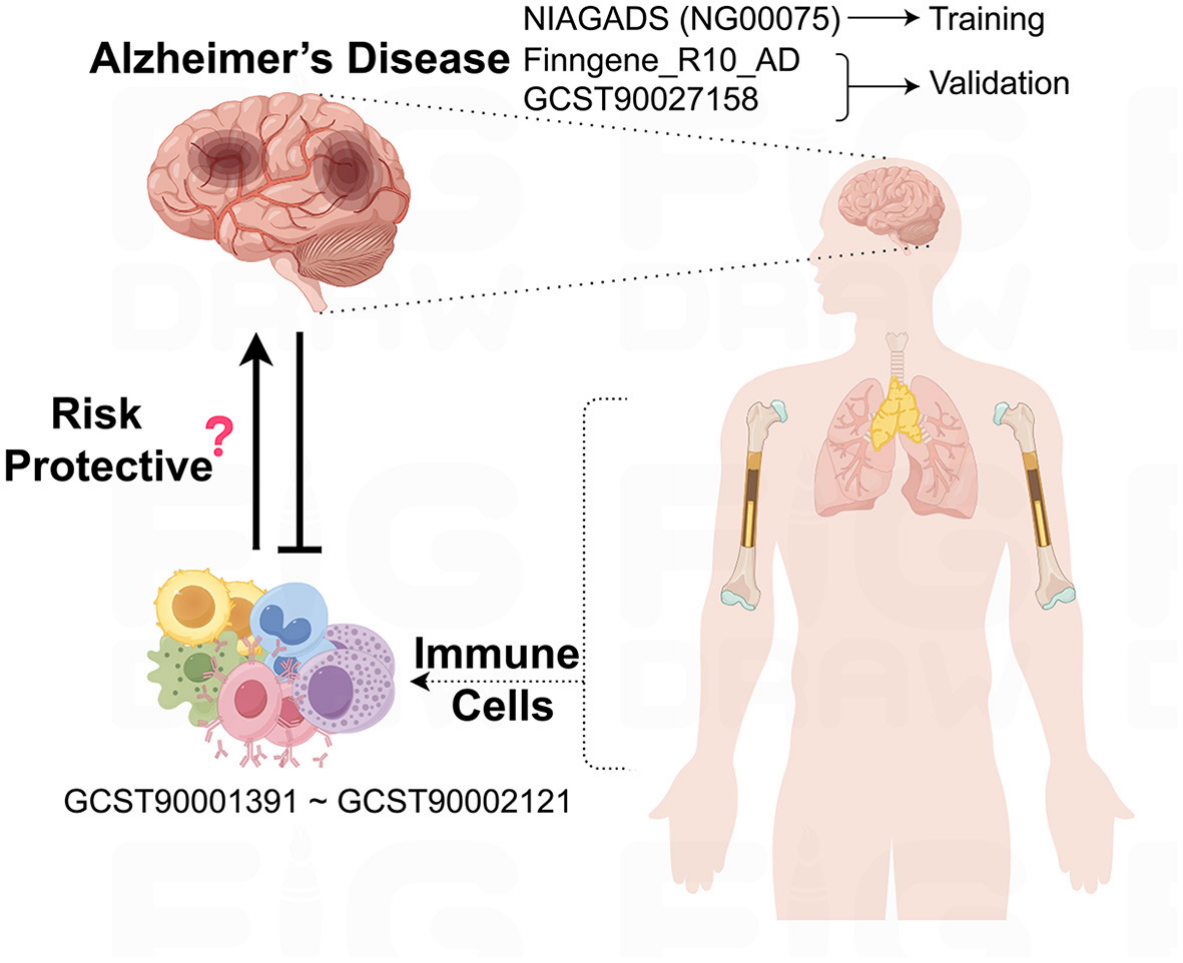
Schematic representation of the study design and data flow. This figure illustrates the process of leveraging publicly available GWAS data to investigate the causal links between immune cell phenotypes and AD.

This study leverages GWAS data sourced from public databases to explore the potential causal relationships between immune cells and AD, as outlined in Figure 1. Before the study's commencement, all pertinent datasets underwent stringent ethical review and were granted approval by the respective institutional review boards, thereby guaranteeing the research's adherence to ethical standards.

### 2.2 Data sources for AD

The underpinning data for this study are derived from European population samples pertaining to AD. We sourced single nucleotide polymorphisms (SNPs) linked to AD from the most extensive GWAS meta-analysis to date, as executed by the National Institute on Aging Genetics of Alzheimer's Disease Data Storage Site (NIAGADS), under the accession code NG00075. This dataset encompasses a total of 35,274 individuals with AD and 59,163 control subjects (Kunkle et al., 2019). Furthermore, SNPs data from the FinnGen database (https://www.finngen.fi/en) were employed for the validation of our research findings, with a total of 10,520 cases and 401,661 controls (Kurki et al., 2023). In addition, we drew upon the summary statistics for AD as cataloged in the GWAS Catalog, identified by the accession number GCST90027158, which includes a total of 39,106 cases and 46,828 controls (Bellenguez et al., 2022). All AD patient samples were collected in strict accordance with established clinical diagnostic standards.

### 2.3 Sources of immunity-spanning GWAS data

The aggregated statistical data for all immunological traits analyzed in this study originate from the GWAS Catalog, with accession numbers spanning from GCST0001391 to GCST0002121. This GWAS investigation included a cohort of 3,757 genetically non-overlapping European individuals, with reference samples derived from the Sardinian population sequence. The study scrutinized approximately 22 million SNPs, conducting correlation tests that were adjusted for covariates such as age, age squared, and gender. Utilizing flow cytometry, a comprehensive analysis of 539 distinct immune cell characteristics was undertaken, encompassing 118 absolute cell counts, 389 median fluorescence intensity values for surface antigens, and 32 morphological parameters. Furthermore, the research integrated 192 relative cell counts, which are the ratios between different cell levels, culminating in the assessment of 731 cellular traits across a population of 3,757 Sardinian residents.

### 2.4 Genetic instrument selection

In our MR analyses, we screened SNPs associated with our exposure of interest at a significance threshold of *p* < 5 × 10^−5^, using these as instrumental variables. To ensure near-independence among these SNPs, we meticulously pruned our selection using a linkage disequilibrium (LD) threshold of r^2^ < 0.1, with a physical distance criterion of 500 kilobase pairs, as implemented in PLINK (Vierstra et al., 2020). In our analysis, we leveraged the Linkage Disequilibrium (LD) reference panel from the 1000 Genomes Project, specifically curated for the European super-population. This panel was meticulously filtered to include only bi-allelic SNPs with a minor allele frequency exceeding 0.01, ensuring a robust genetic representation for our study. The F statistic, a measure widely utilized in the context of instrumental variable regression models to assess the strength of the instrumental variable, was calculated for each SNP. Subsequently, SNPs with an F statistic value below 10 were excluded from the analysis to mitigate the risk of weak instruments bias, as recommended in the literature (Burgess et al., 2011).

### 2.5 Statistical analyses

For this study, we employed R software (version 4.3.3) to perform comprehensive statistical analyses aimed at discerning the causal relationship between 731 distinct immune phenotypes and AD. Our analytical approach encompassed a suite of robust statistical techniques, including MR-Egger regression, the weighted median method, weighted mode analysis, the sample mode approach, and inverse variance weighted analysis. All these analyses were executed using the “TwoSampleMR” R package, which facilitated a streamlined workflow. To assess instrumental heterogeneity across variables, we applied Cochran's Q statistic in tandem with the horizontal multidimensional MR-Egger method. A significant intercept term in this method indicates the presence of heterogeneity. Upon identifying and excluding SNPs that contributed to this heterogeneity, we re-conducted the inverse variance weighted analysis to refine our results. Additionally, we conducted a targeted search for SNPs with suggestive associations (*p* < 1 × 10^−5^) relative to these risk factors, utilizing the Phenoscanner V2 platform. This allowed us to further explore potential genetic markers linked to AD. Finally, our visual assessment through a scatter plot indicated that outliers exert minimal influence on the dataset. Meanwhile, the funnel plot revealed a strong correlation among the results, with no significant heterogeneity observed.

## 3 Results

### 3.1 The causal relationship between immunophenotypic traits and AD

In a meticulous examination of the possible causal dynamics interconnecting immune cells and the initiation as well as the escalation of AD, we engaged a diverse array of statistical techniques to fortify the integrity and precision of our investigation. To elaborate, we harnessed five discrete yet methodologically robust approaches: the Inverse Variance Weighted, the MR Egger, the Weighted Median, the Weighted Mode, and the Simple Mode. Within this analytical arsenal, the Inverse Variance Weighted method emerged as the cornerstone of our methodology, selected for its exceptional statistical robustness and its demonstrated efficacy in providing stable and reliable estimates across a wide range of research contexts.

**Figure 2:**
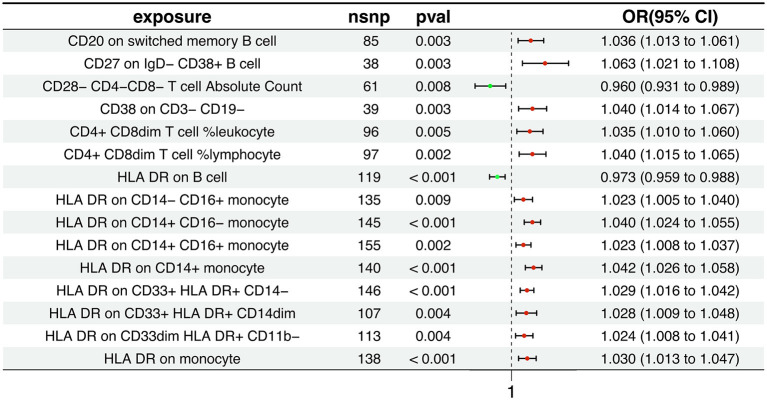
Association between immunophenotypes and AD risk. The figure displays the results from the Inverse Variance Weighted analysis, highlighting the protective effect of two immunophenotypes: the absolute count of CD28^−^CD4^−^CD8^−^ T cells and the expression of HLA DR on B cells. OR, 95% CI, and *p*-values are provided for each association.

Our research findings suggest that 15 distinct immune cell types are involved in the pathogenesis of AD. Notably, two immunophenotypes appear to exert a protective effect against AD: the absolute count of CD28^−^CD4^−^CD8^−^ T cells and the expression of HLA DR on B cells, as depicted in Figure 2. Using the Inverse Variance Weighted method, we evaluated the association between these immunophenotypes and AD risk. For CD28^−^CD4^−^CD8^−^ T cells, we found an odds ratio (OR) of 0.960 (95% Confidence Interval [CI] 0.931-0.989, *p* = 0.008). Similarly, HLA DR on B cells was associated with a reduced risk, with an OR of 0.973 (95% CI 0.959-0.988, *p* < 0.001). In contrast, the remaining 13 immunophenotypes were identified as risk factors for AD, with varying OR and 95% CI, as detailed in Supplementary Table S1. The MR-Egger regression intercept provided evidence against the potential for horizontal pleiotropy, reinforcing the reliability of our causal inference, as shown in Supplementary Table S2. Furthermore, visual assessments such as the funnel plot, scatter plot, forest plot, and a leave-one-out analysis consistently demonstrated the stability and reliability of our findings from multiple analytical perspectives (Supplementary Figures S1, S2). These rigorous diagnostic checks enhance the credibility of our study's conclusions and strengthen the confidence in the observed immunological relationships with AD.

### 3.2 The causal relationship between AD and immunophenotypic traits

**Figure 3:**
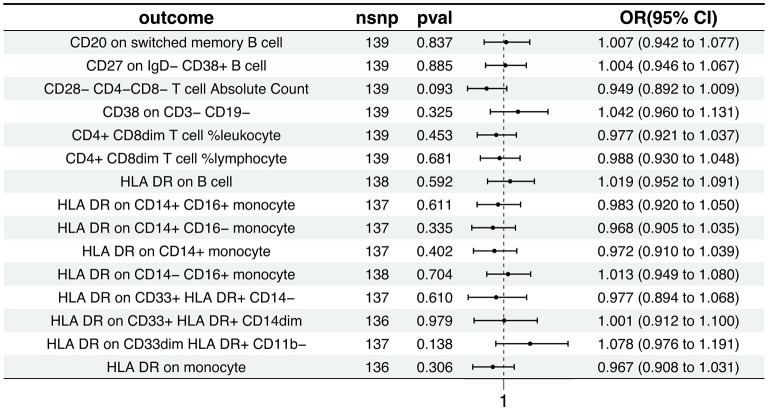
Causal effects of AD on immunophenotypic traits. This figure presents the Inverse Variance Weighted analysis outcomes, indicating a predominantly association between AD and various immunophenotypic traits. The graphical representation includes OR, 95% CI, and *p*-values for each trait, demonstrating the lack of statistically significant effects.

The Inverse Variance Weighted analysis revealed a negative association between AD and several immunophenotypic traits. For instance, the OR and 95% CI for the association with AD were as follows: CD20 on switched memory B cell (OR = 1.007, 95% CI = 0.942–1.078, *p* = 0.837), CD27 on IgD^−^ CD38^+^ B cell (OR = 1.004, 95% CI = 0.946–1.067, *p* = 0.885), and so on for the remaining traits. Notably, all associations had *p* > 0.05, indicating no statistically significant effect. The MR analysis further explored the causal effects of AD on these immunophenotypic traits, with results depicted in Figure 3 and detailed in Supplementary Tables S3, S4. Additional visual assessments, including scatter plots and forest plots, are provided in Supplementary Figures S3, S4.

### 3.3 Validation of between immunophenotypic traits and AD

To further validate the causal relationship between immunophenotypic traits and the onset and progression of AD, we selected two distinct sets of AD data for verification: the FinnGen cohort and the dataset identified as GCST90027158.

**Figure 4:**
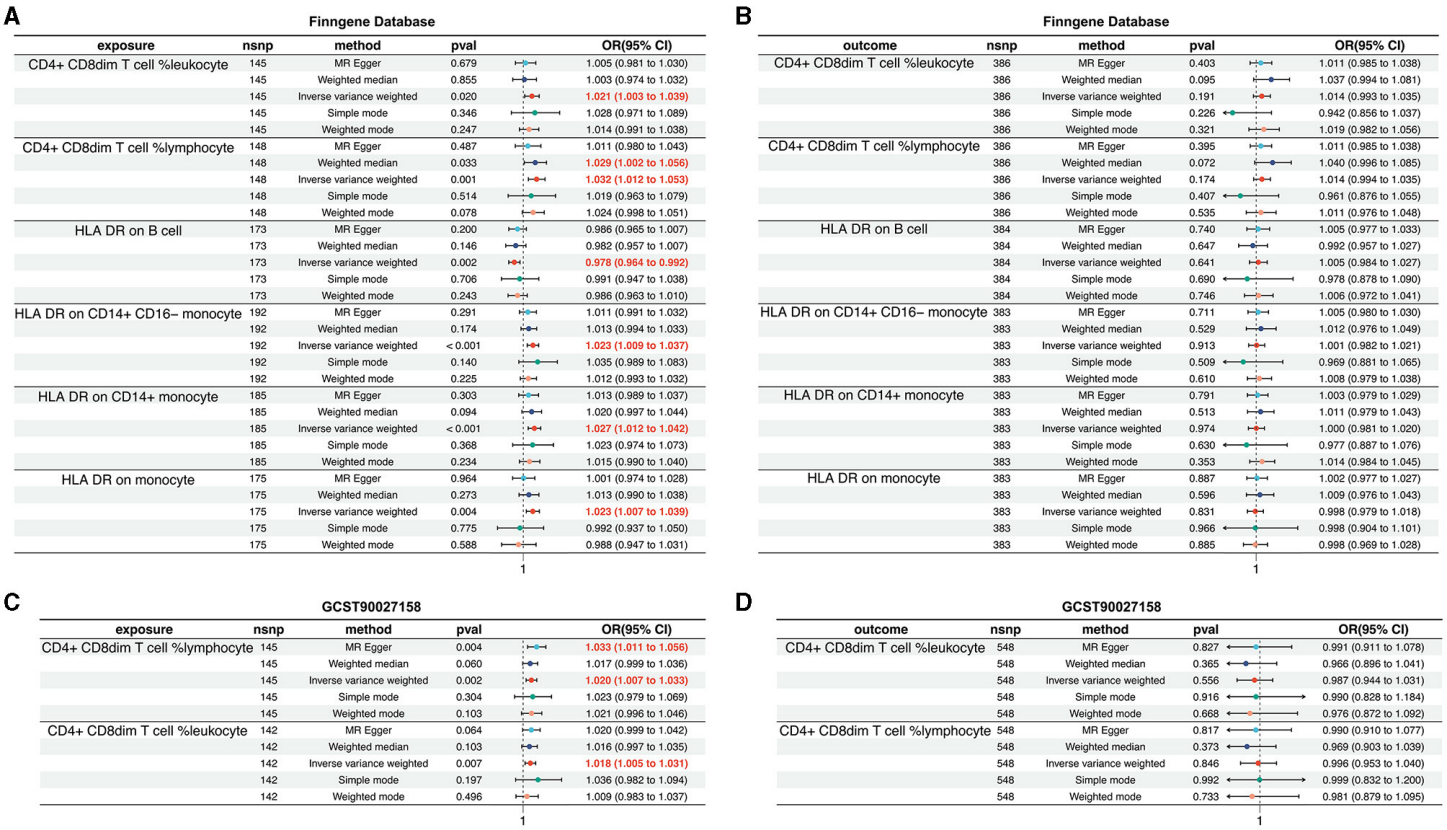
Validation of the causal relationship between immunophenotypic traits and AD in the FinnGen and GCST90027158 datasets. **(A, B)** depict the confirmed causal associations with specific immune cell types, including CD4^+^CD8^dim^ T cell leukocyte and lymphocyte, and HLA DR expression on B cells and monocytes within the FinnGen dataset. **(C, D)** show the causal relationships for the immunophenotypic traits analyzed in the GCST90027158.

Within the FinnGen dataset, we identified a causal relationship between AD and six specific immune cell types. These include CD4^+^CD8^dim^ T cell leukocyte (OR = 1.021, 95%CI = 1.003–1.039, *p* = 0.020), CD4^+^CD8^dim^ T cell lymphocyte (OR = 1.032, 95% CI = 1.012–1.053, *p* = 0.001), HLA DR on B cell (OR = 0.978, 95% CI = 0.964–0.992, *p* = 0.002), HLA DR on CD14^+^CD16^−^ monocyte (OR = 1.023, 95% CI = 1.009–1.037, *p* < 0.001), HLA DR on CD14^+^ monocyte (OR = 1.027, 95% CI = 1.012–1.042, *p* < 0.001), and HLA DR on monocyte (OR = 1.023, 95% CI = 1.007–1.039, *p* = 0.004). These findings are illustrated in Figure 4A, with additional details provided in Supplementary Figures S5, S6, Supplementary Tables S5, S6. However, the analysis of the same FinnGen dataset did not reveal a causal relationship for the remaining immunophenotypic traits (Figure 4B, Supplementary Figures S7, S8, Supplementary Table S7).

In the GCST90027158 dataset, a causal relationship was found between two specific immune cell types and the development of AD: the CD4^+^ CD8^dim^ T cell leukocyte (OR = 1.020, 95% CI = 1.007–1.033, *p* = 0.002) and the CD4^+^ CD8^dim^ T cell lymphocyte (OR = 1.018, 95% CI = 1.005–1.031, *p* = 0.007). These results are presented in Figure 4C, with further details in Supplementary Figures S9, S10, Supplementary Tables S8, S9. However, no causal link was established between AD and other immunophenotypic traits (Figure 4D, Supplementary Figures S11, S12, Supplementary Table S10).

## 4 Discussion

Within the pathogenesis of AD, the roles of the immune system are becoming increasingly recognized. Utilizing publicly accessible GWAS data, this study conducted an exhaustive analysis to elucidate potential causal links between a spectrum of immune cell phenotypes and AD. This study examined a range of immune cell phenotypes, including CD4^+^CD8^dim^ T cell leukocytes, CD4^+^CD8^dim^ T cell lymphocytes, B cells expressing HLA DR, CD14^+^CD16^−^ monocytes, CD14^+^ monocytes, and monocytes in general. Our research has not only confirmed the connection between AD and the immune system but also identified specific immune cell characteristics that may be key to early detection, disease monitoring, and the development of targeted treatments. Furthermore, these findings guide the direction of future research and clinical approaches.

During the maturation of T cells within the human immune system, CD4 and CD8 molecules are typically not found together on the surface of a single cell. These molecules distinguish the two primary subpopulations of T cells: CD4^+^ T cells and CD8^+^ T cells. Notably, the Th1 and Th17 subsets of CD4^+^ T cells are capable of secreting various pro-inflammatory cytokines, such as interferon-gamma (IFN-γ) and tumor necrosis factor-alpha (TNF-α). These cytokines have the ability to activate microglia and intensify neuroinflammation, which significantly influences the pathogenesis of AD (Anderson et al., 2014; Machhi et al., 2021; Chen et al., 2023). Microglia are the primary immune cells of the central nervous system, and their activation in AD can promote inflammatory responses, which are associated with the formation of Aβ plaques and neurodegeneration (DeMaio et al., 2022). IFNγ, as an important cytokine, can enhance the activation state of microglia, leading to the release of inflammatory mediators such as TNF-α, interleukin-1 beta (IL-1β), and nitric oxide, all of which may cause neuronal damage and cognitive decline (Glass et al., 2010). IFNγ may also promote the entry of peripheral immune cells into the brain by altering the permeability of the blood-brain barrier, further exacerbating neuroinflammation. This inflammatory environment not only affects the clearance of Aβ but may also interfere with the normal function of tau protein, leading to its abnormal phosphorylation and aggregation, forming neurofibrillary tangles, and further intensifying the onset and progression of AD (Glass et al., 2010). Furthermore, the observation of clonal expansion of CD8^+^ T cells in the cerebrospinal fluid of AD patients implies that they may have a pivotal role in the immune response within the central nervous system (Gate et al., 2020). Neuroinflammation is a critical pathological process in the brains of AD patients (Leng and Edison, 2021). Both CD4^+^ and CD8^+^ T cells have the capacity to traverse the blood-brain barrier, thereby gaining entry to the brain and engaging in the modulation of central neuroinflammation, which in turn affects the progression of AD (Jorfi et al., 2023b). In the context of AD pathology, there is a marked increase in the infiltration of CD4^+^ T cells, CD8^+^ T cells, and monocytes within brain models, which is likely closely associated with neurologic damage related to AD (Jorfi et al., 2023b). Beyond these two predominant T cell types, researchers have identified a minor subset of T cells exhibiting a double-positive CD4^+^CD8^dim^ phenotype, which constitutes a small fraction of the CD3^+^ T cell pool (Suni et al., 2001). Studies have shown that CD4^+^CD8^dim^ T cells possess a more potent specific cytotoxic capability against cytomegalovirus (CMV) compared to conventional CD4^+^ T cells (Suni et al., 2001). Additionally, CD4^+^CD8^dim^ T cells, which originate from double-positive T cells, express CD13, a molecule that conveys signals for antigen stimulation and may thus participate in the regulation of immune responses (Sala et al., 1993; Sun et al., 2023a). As a result, CD4^+^CD8^dim^ T cells could potentially serve dual roles by both assisting and directly killing in the immune response. While there are no current reports on the role of CD4^+^CD8^dim^ T cells in the etiology of AD, our research suggests that these cells may have a significant risk association with the development of AD. Consequently, reducing the population of CD4^+^CD8^dim^ T cells could be crucial in mitigating neuroinflammation within the brains of AD patients and potentially slowing the onset of the disease.

The HLA DR molecules, an essential element of the major histocompatibility complex class II (MHC-II), are proficiently displayed on the B lymphocyte cell surface, where they act as a pivotal nexus facilitating the activation of these cells. Moreover, HLA DR molecules on B cells play an instrumental role in presenting antigenic peptides to CD4^+^ T cells, also known as helper T cells. This interaction activates T cells, which then stimulate the proliferation and differentiation of B cells, ultimately resulting in antibody production (Celis-Giraldo et al., 2024; Paterson et al., 2024). Notably, B cells may intensify neuroinflammation in AD by promoting T cell infiltration (Griffith et al., 2022; Abualrous et al., 2023; Chen et al., 2023). Once T cells cross the blood-brain barrier and enter the central nervous system (CNS), they can release a range of inflammatory mediators, including cytokines and chemokines (Cao and Zheng, 2018; Wu et al., 2021). These mediators not only recruit more immune cells but may also exacerbate local inflammatory responses. As inflammation persists, neurons may suffer damage or even die, thereby accelerating the onset and progression of AD (Cao and Zheng, 2018; Wu et al., 2021). In contrast, the interaction between T cells activated by B cells and microglia, the primary immune cells in the CNS, may lead to the activation of microglia. Microglia play a key role in clearing Aβ plaques and regulating neuroinflammation (Jorfi et al., 2023a; Zhang et al., 2023). This activation may help maintain the stability of the brain's internal environment, but during the pathological process of AD, it may also cause damage to neurons due to excessive or dysregulated immune responses (Jorfi et al., 2023a; Zhang et al., 2023). Therefore, the interaction between T cells and microglia plays a complex role in AD, as they can both promote neuroprotection and exacerbate neurodegeneration. Understanding the delicate balance between these cells is crucial for developing immune-modulating therapeutic strategies for AD. B cells are pivotal in humoral immunity, with the ability to differentiate into plasma cells that generate antibodies tailored to specific antigens. In the context of AD, these antibodies can bind to Aβ, thereby aiding in its removal (Kim et al., 2021). Additionally, the interaction between B cell-derived antibodies and the transmembrane protein TREM2 on microglia significantly influences microglial functionality and the clearance of Aβ plaques (Zhao et al., 2022). Studies have shown a correlation between a reduction in B cell count in the peripheral blood of AD patients and the severity of their condition (Kim et al., 2021; Xiong et al., 2021). Mouse model experiments have demonstrated that B cell depletion can exacerbate cognitive impairment in AD models and lead to a substantial buildup of Aβ in the brain (Kim et al., 2021; Xiong et al., 2021). Our research indicates that HLA DR on B cells may offer significant protection against the onset and progression of AD. Consequently, modulating B cell activity or the antibodies they produce could emerge as a promising therapeutic approach for AD. This insight paves the way for novel avenues in therapeutic research, potentially leading to the development of new treatments that target B cells or their antibodies, with the goal of mitigating or arresting the progression of AD.

Monocytes, the most sizable leukocytes in the circulatory system, possess the capacity to differentiate into macrophages and dendritic cells of monocyte origin. These cells, along with their progeny, play pivotal roles in modulating inflammatory responses and facilitating tissue repair, which are likely critical to the pathogenesis of AD (Sun et al., 2023b). Monocytes are categorized into three discrete subpopulations, delineated by their differential expression of CD14 and CD16 surface markers: classical monocytes (CD14^+^CD16^−^), intermediate monocytes (CD14^+^CD16^+^), and non-classical monocytes (CD14^−^CD16^+^) (Ziegler-Heitbrock et al., 2010). The CD14^+^ subset of monocytes is notably involved in the recognition of bacteria and the subsequent immune response, facilitated by their interaction with lipopolysaccharide through CD14 molecules (Draude et al., 1999; Wu et al., 2019). CD16, also known as FcγRIII, serves as a receptor for the Fc fragment of immunoglobulin G and is primarily associated with the function of natural killer cells (Feng et al., 2021). Classical monocytes are especially significant in orchestrating inflammatory responses and in the presentation of antigens (Serbina et al., 2008). Upon infiltrating the brain, these cells mature into microglia, playing an essential part in neuroinflammatory processes and the clearance of Aβ associated with AD (Habib et al., 2020). Furthermore, classical monocytes have the ability to express MHC-II molecules, allowing them to present antigens to CD4^+^ T cells. This potential to drive T cell infiltration and activation may contribute to the exacerbation of neuroinflammation in AD (Keren-Shaul et al., 2017; Simren et al., 2021). Our research has established a correlation between the expression of HLA DR on monocytes, specifically HLA DR on CD14^+^ monocytes and HLA DR on the CD14^+^CD16^−^ subset, and an increased risk of developing AD. Given the manifold functions of monocytes within the AD pathology, they could present promising targets for therapeutic approaches. Interventions aimed at modulating the activity or differentiation pathways of monocytes may yield considerable therapeutic advantages in the clinical management of AD.

Utilizing a bidirectional MR strategy, this study conducted an exhaustive analysis of genomic data from 94,437 individuals with AD. The primary goal was to elucidate the causal relationship between AD and immune cells, with a focus on ensuring the precision and wide-ranging applicability of the statistical results. The research findings are grounded in genetic instrumental variables and feature a suite of robust MR analytical methods, meticulously designed to mitigate the influence of horizontal pleiotropy and other confounding factors. Moreover, the findings have been substantiated through cross-validation with multiple independent datasets, thereby reinforcing the reliability and validity of the study's conclusions. However, this analytical method still has its limitations. MR analysis primarily relies on common genetic variations and may underestimate the potential impact of rare genetic variations on disease risk. MR analysis may be affected by confounding factors, although we have employed various methods to mitigate this risk. The data for this study were all derived from European population samples, lacking statistical results from non-European populations, which makes the findings less generalizable. Therefore, this is also a main direction for our future research.

While providing substantial insights, this study faces certain limitations. First and foremost, its foundation on a database that is heavily skewed toward European populations may limit the broad applicability of its conclusions across different ethnic groups. Additionally, the lack of detailed individual-level data poses a challenge in thoroughly assessing the intricate interplay between genetic and environmental factors, which could, in turn, affect the precision of establishing causal relationships. Furthermore, the Mendelian Randomization (MR) analysis, which primarily focuses on common genetic variants, risks underestimating the significant impact that rare genetic variations might have on disease risk, possibly indicating a gap in the current analytical approach. Lastly, although MR analysis presents an observational data-driven method to investigate the causal link between immune cells and Alzheimer's disease, the robustness of its conclusions would benefit from further substantiation through rigorous experimental research.

## 5 Conclusion

In conclusion, our thorough a bidirectional MR analysis has exposed the causal ties linking a spectrum of immunophenotypes to AD, and has shed light on the sophisticated interplay between the immune system and AD. Importantly, our research has effectively minimized the confounding effects of reverse causality, extraneous variables, and other uncontrollable confounding factors. This has equipped researchers with a novel vantage point from which to delve into the intricate biological foundations of AD. Our findings not only hold the potential to inform the creation of early intervention and therapeutic strategies but also contribute to the expansion of the field of psychoneuroimmunology. They provide significant insights that are instrumental for the prevention and management of AD.

## Data availability statement

The original contributions presented in the study are included in the article/Supplementary material, further inquiries can be directed to the corresponding authors.

## Ethics statement

The data for this study were obtained solely from publicly available datasets, and have been diligently reviewed the database usage guidelines. Given the nature of the data and the scope of the analysis, no ethical approval was required for this research.

## Author contributions

EZ: Conceptualization, Investigation, Project administration, Writing – original draft, Writing – review & editing. TC: Conceptualization, Visualization, Writing – review & editing. YC: Data curation, Writing – review & editing. CL: Conceptualization, Writing – review & editing. LT: Conceptualization, Data curation, Writing – review & editing. XS: Writing – review & editing. FD: Project administration, Writing – review & editing.

## Supplementary material

Supplementary Figure S1 Funnel plot assessing publication bias in the MR analysis of immunophenotypic traits as exposures and AD as the outcome. The plot visualizes the estimated effect size (β_IV_) against the inverse of the standard error (1/SE_IV_), with asymmetry potentially indicating publication bias.

Supplementary Figure S2 Scatter plots depict the results of MR tests for various immunophenotypic traits as exposures against AD as the outcome. The scatter plots illustrate the relationship between the effect of SNPs on the exposure and their corresponding effect on the outcome, allowing for the assessment of potential causal relationships.

Supplementary Figure S3 Funnel plot designed to assess publication bias in MR analysis where AD is the exposure and various immunophenotypic traits are the outcomes.

Supplementary Figure S4 Scatter plots depict the results of MR tests for AD as exposures against various immunophenotypic traits as the outcome.

Supplementary Figure S5 Funnel plot for assessing publication bias in MR analysis specific to the FinnGen dataset, with immunophenotypic traits as exposures and AD as the outcome.

Supplementary Figure S6 Scatter plot for FinnGen dataset, illustrating MR effect sizes for immunological exposures on AD outcome.

Supplementary Figure S7 Funnel plot for assessing publication bias in MR analysis of the FinnGen dataset, with AD as the exposure and immunophenotypic traits as outcomes.

Supplementary Figure S8 Scatter plot for FinnGen dataset, presenting MR effect sizes for AD exposure on immunological outcomes.

Supplementary Figure S9 Funnel plot for assessing publication bias in MR analysis specific to the GCST90027158 dataset, with immunophenotypic traits as exposures and AD as the outcome.

Supplementary Figure S10 Scatter plot for GCST90027158 dataset, displaying MR effect sizes for immunological exposures on AD outcome.

Supplementary Figure S11 Funnel plot for assessing publication bias in MR analysis of the GCST90027158 dataset, with AD as the exposure and immunophenotypic traits as outcomes.

Supplementary Figure S12 Scatter plot for GCST90027158 dataset, presenting MR effect sizes for AD exposure on immunological outcomes.

Supplementary Table S1 The Causal of immunophenotypic traits to Alzheimer's disease (NG00075).

Supplementary Table S2 MR result of pleiotropy test for immune cells to AD (NG00075).

Supplementary Table S3 The causal of Alzheimer's disease (NG00075) to immunophenotypic traits.

Supplementary Table S4 MR result of pleiotropy test for AD (NG00075) to immune cells.

Supplementary Table S5 The causal of immunophenotypic traits to Alzheimer's disease (FinnGen database).

Supplementary Table S6 MR result of pleiotropy test from immune cell t0 AD (FinnGen database).

Supplementary Table S7 MR result of pleiotropy test from AD (FinnGen database) to immune cells.

Supplementary Table S8 The causal of immunophenotypic traits to Alzheimer's disease (GCST90027158).

Supplementary Table S9 MR result of pleiotropy test from immune cells to AD (GCST90027158).

Supplementary Table S10 MR result of pleiotropy test from AD (GCST90027158) to immune cells.
