# Supplementary figures and images for: The role of Immune cells in Alzheimer's disease: a bidirectional Mendelian randomization study
Source: Front Aging Neurosci. 2024 Jul 15;16:1433691. doi: 10.3389/fnagi.2024.1433691 (PMC11284151; doi:10.3389/fnagi.2024.1433691)

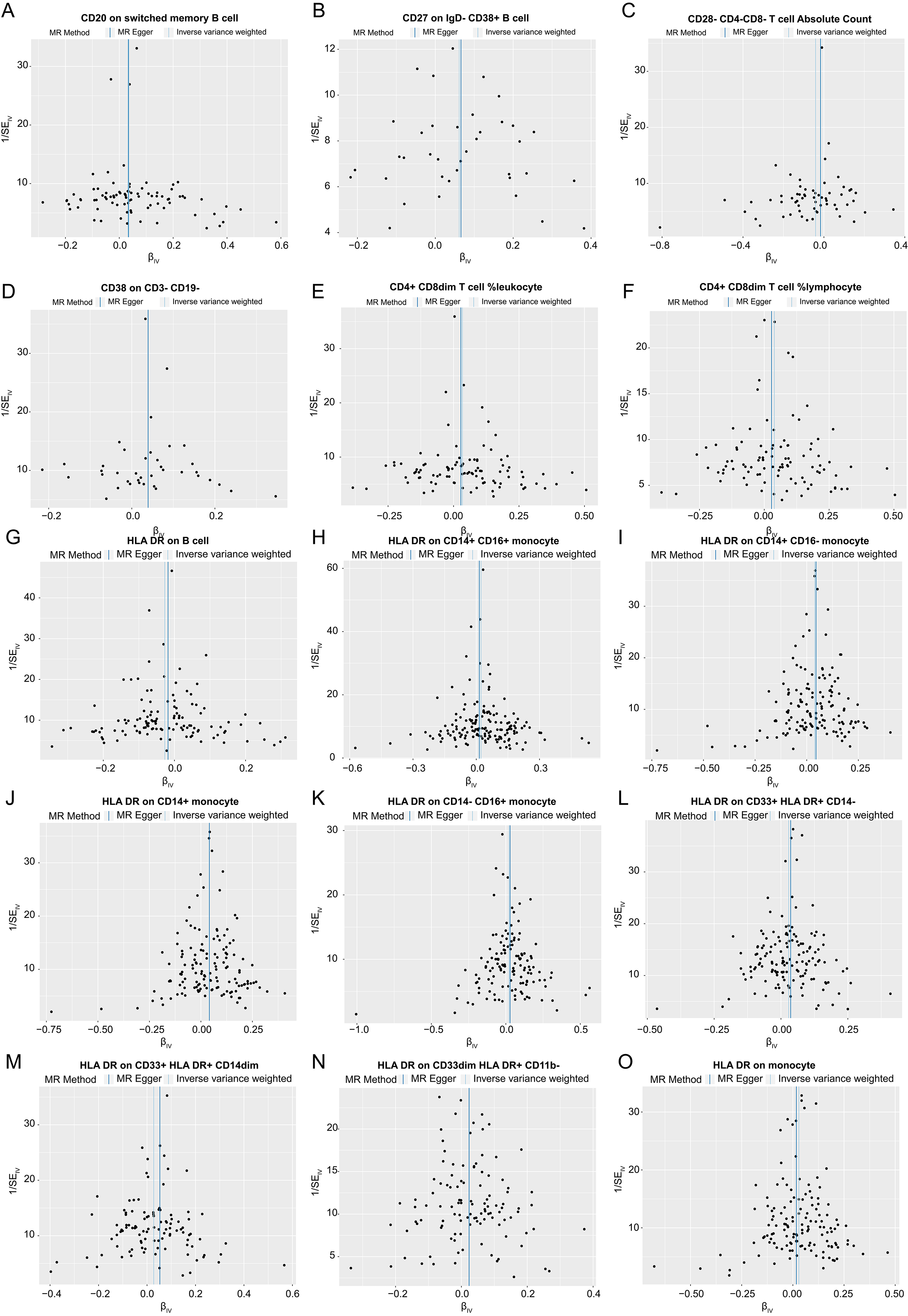

Supplement: Supplementary Figure S1 — Funnel plot assessing publication bias in the MR analysis of immunophenotypic traits as exposures and AD as the outcome. The plot visualizes the estimated effect size (βIV) against the inverse of the standard error (1/SEIV), with asymmetry potentially indicating publication bias. [file Image_1.TIF]

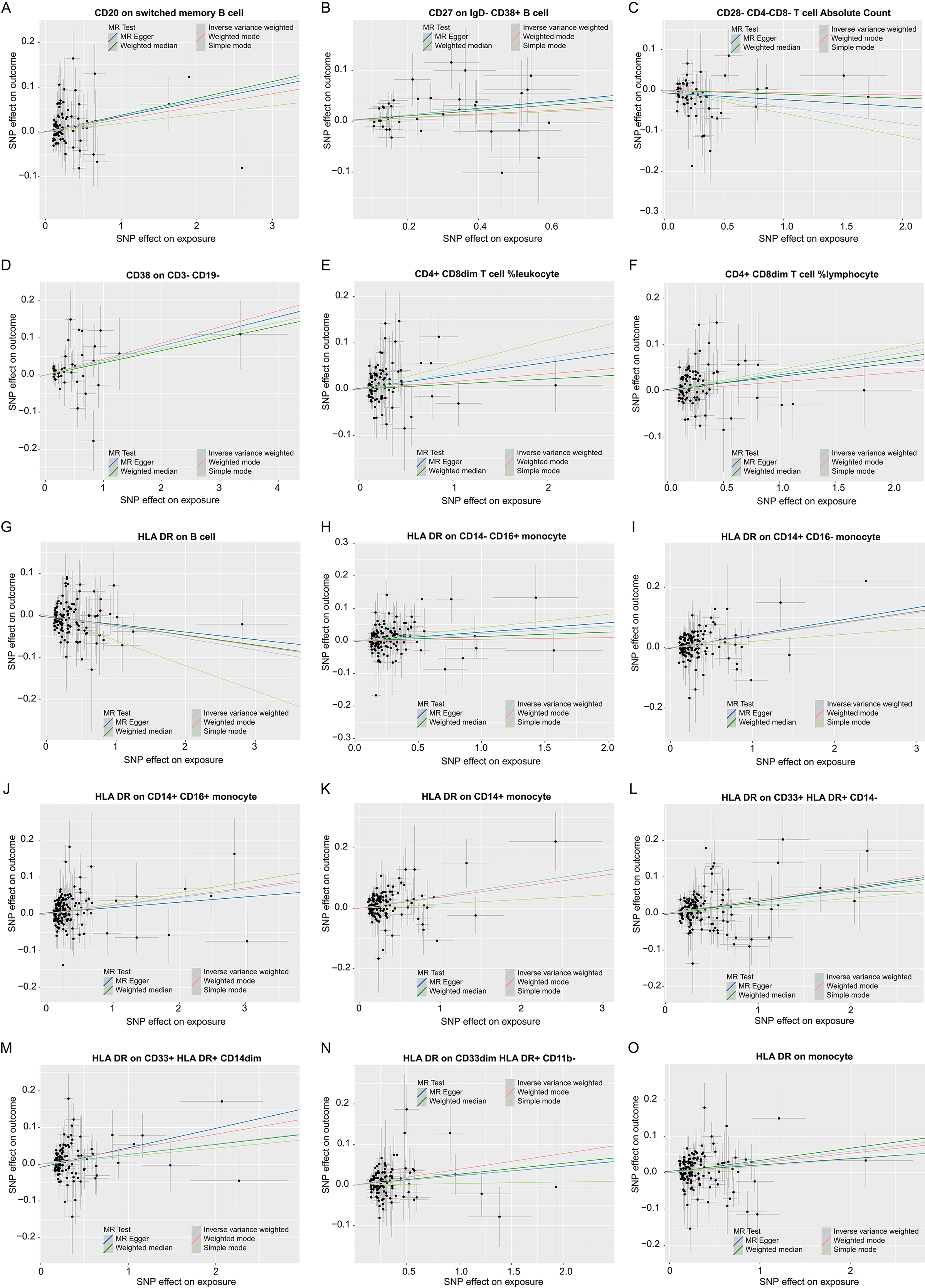

Supplement: Supplementary Figure S2 — Scatter plots depict the results of MR tests for various immunophenotypic traits as exposures against AD as the outcome. The scatter plots illustrate the relationship between the effect of SNPs on the exposure and their corresponding effect on the outcome, allowing for the assessment of potential causal relationships. [file Image_2.TIF]

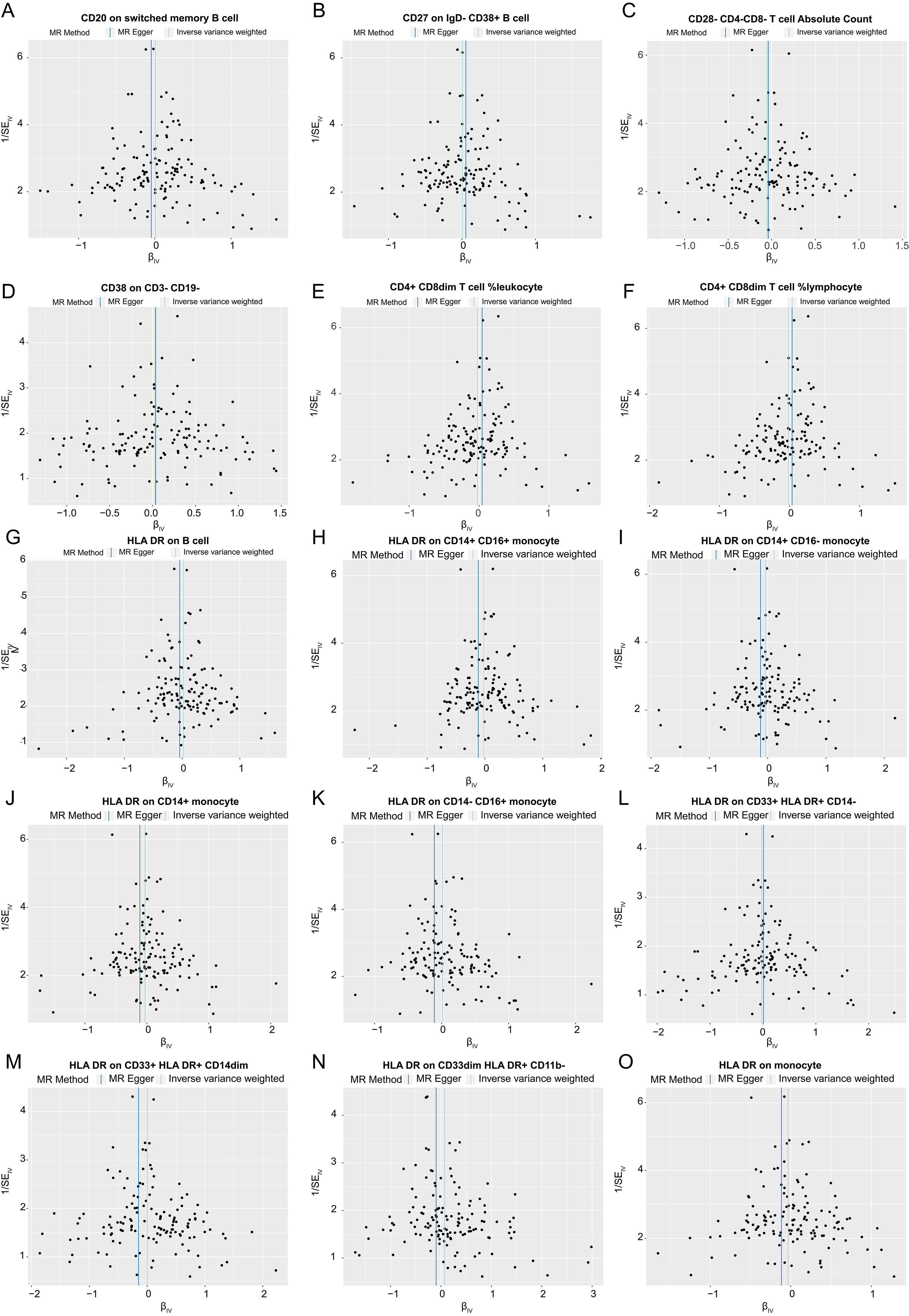

Supplement: Supplementary Figure S3 — Funnel plot designed to assess publication bias in MR analysis where AD is the exposure and various immunophenotypic traits are the outcomes. [file Image_3.TIF]

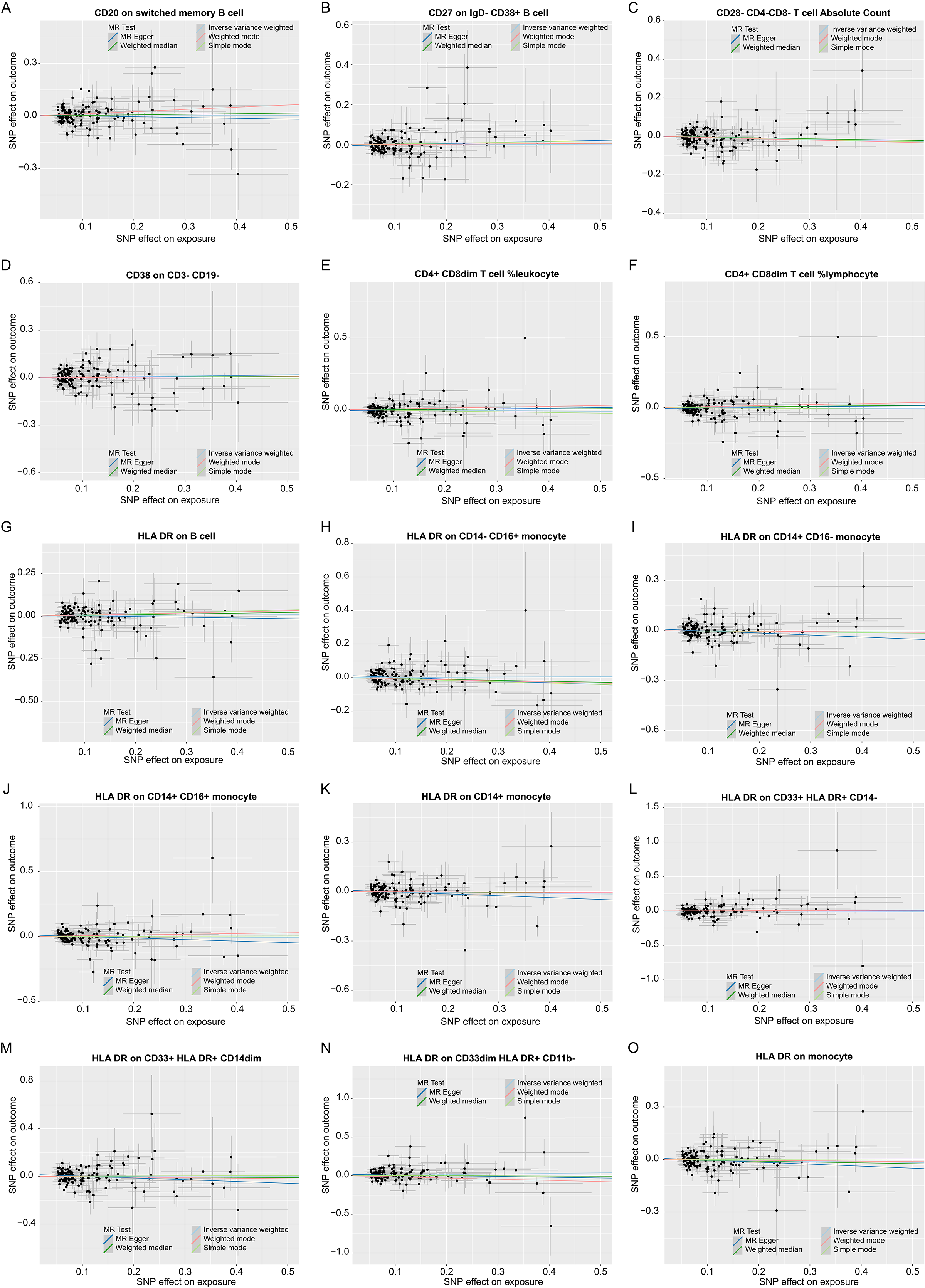

Supplement: Supplementary Figure S4 — Scatter plots depict the results of MR tests for AD as exposures against various immunophenotypic traits as the outcome. [file Image_4.TIF]

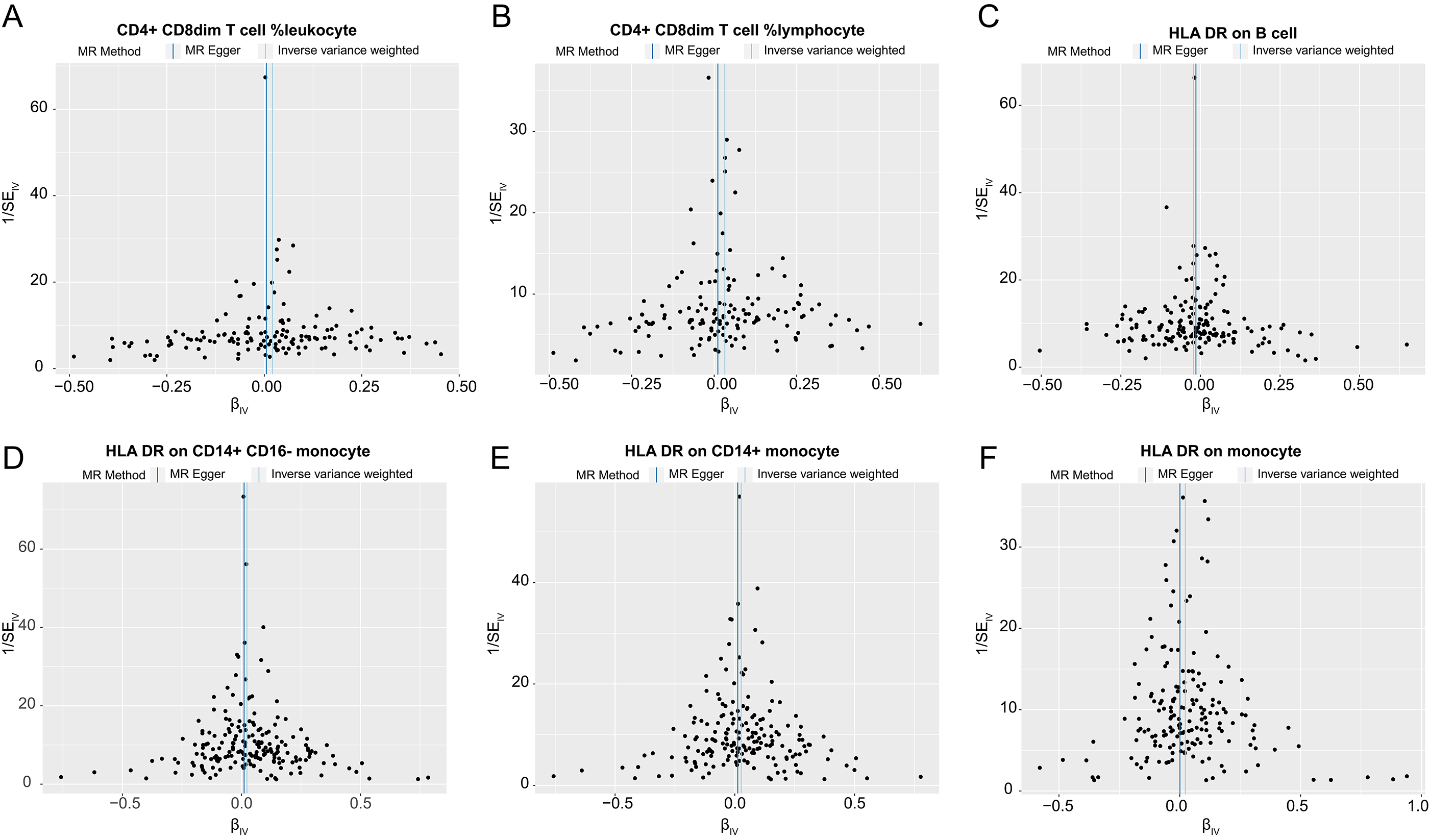

Supplement: Supplementary Figure S5 — Funnel plot for assessing publication bias in MR analysis specific to the FinnGen dataset, with immunophenotypic traits as exposures and AD as the outcome. [file Image_5.TIF]

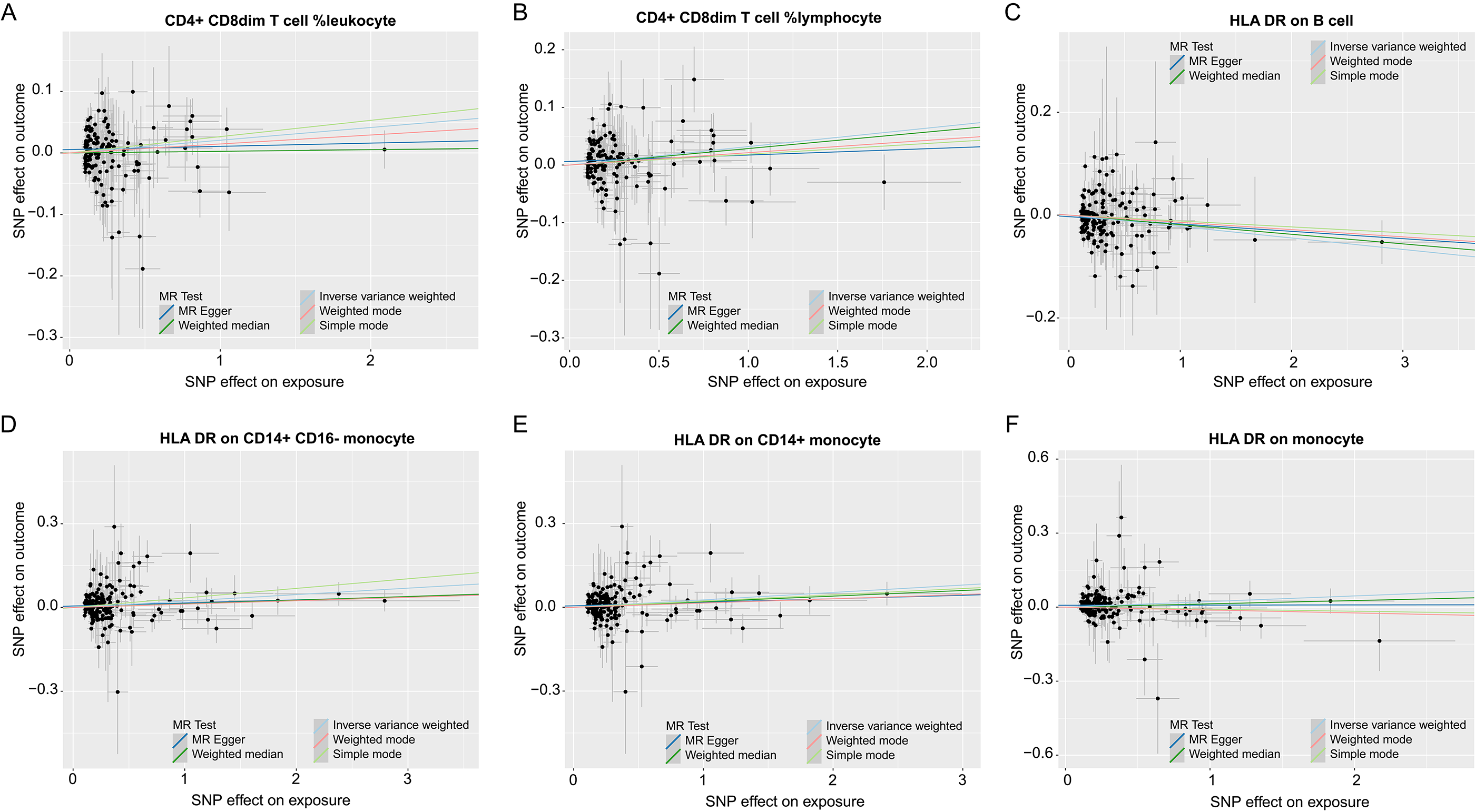

Supplement: Supplementary Figure S6 — Scatter plot for FinnGen dataset, illustrating MR effect sizes for immunological exposures on AD outcome. [file Image_6.TIF]

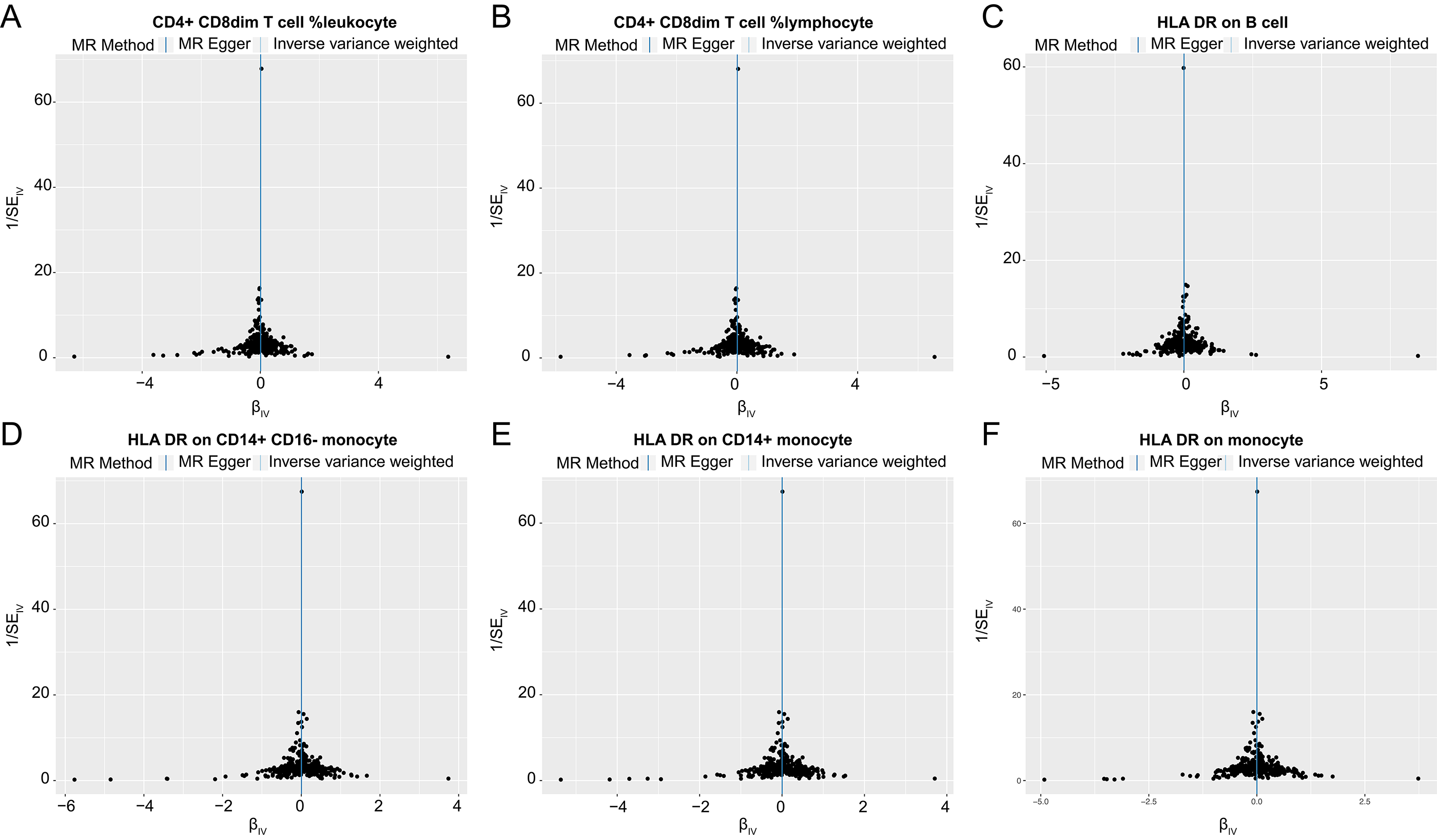

Supplement: Supplementary Figure S7 — Funnel plot for assessing publication bias in MR analysis of the FinnGen dataset, with AD as the exposure and immunophenotypic traits as outcomes. [file Image_7.TIF]

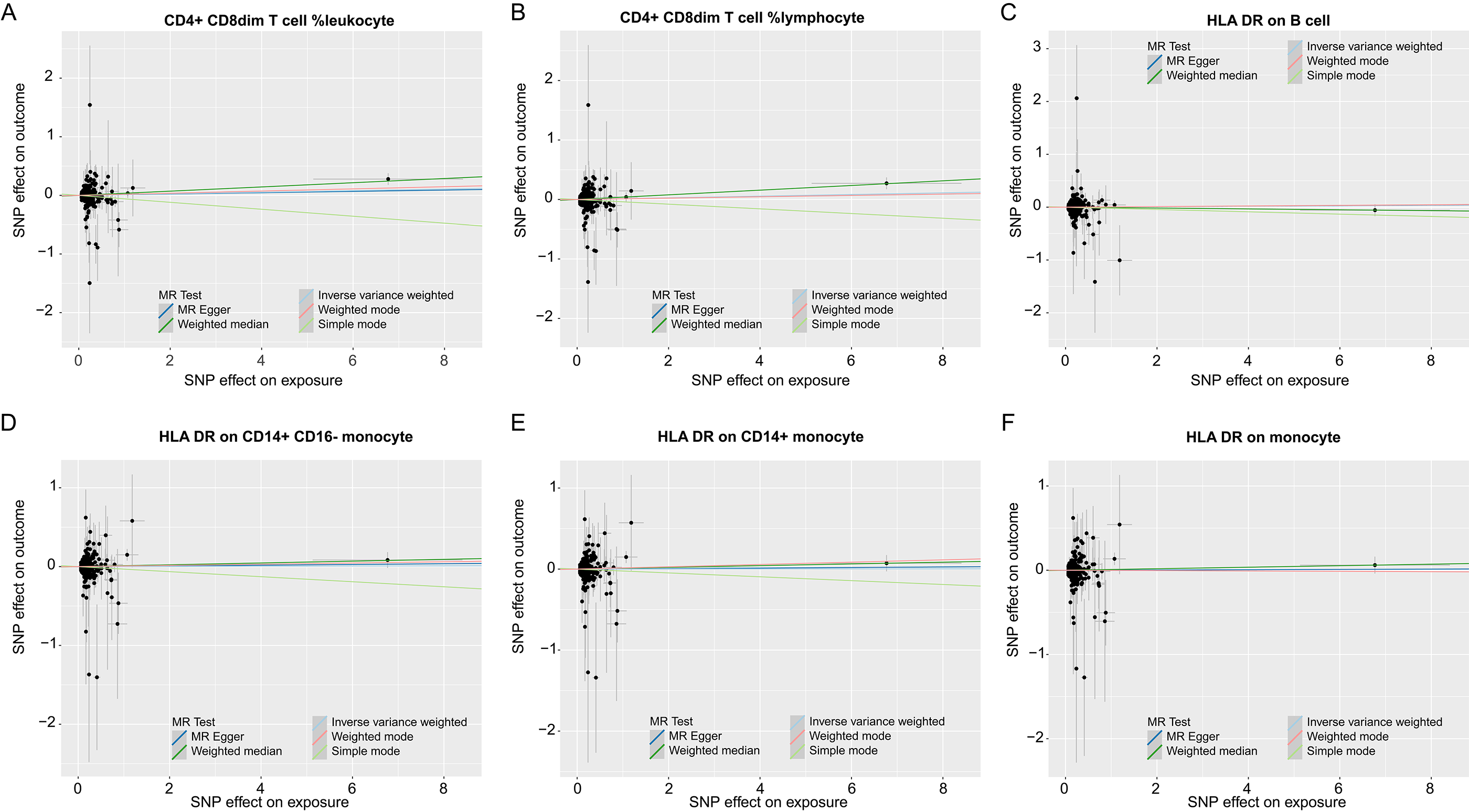

Supplement: Supplementary Figure S8 — Scatter plot for FinnGen dataset, presenting MR effect sizes for AD exposure on immunological outcomes. [file Image_8.TIF]

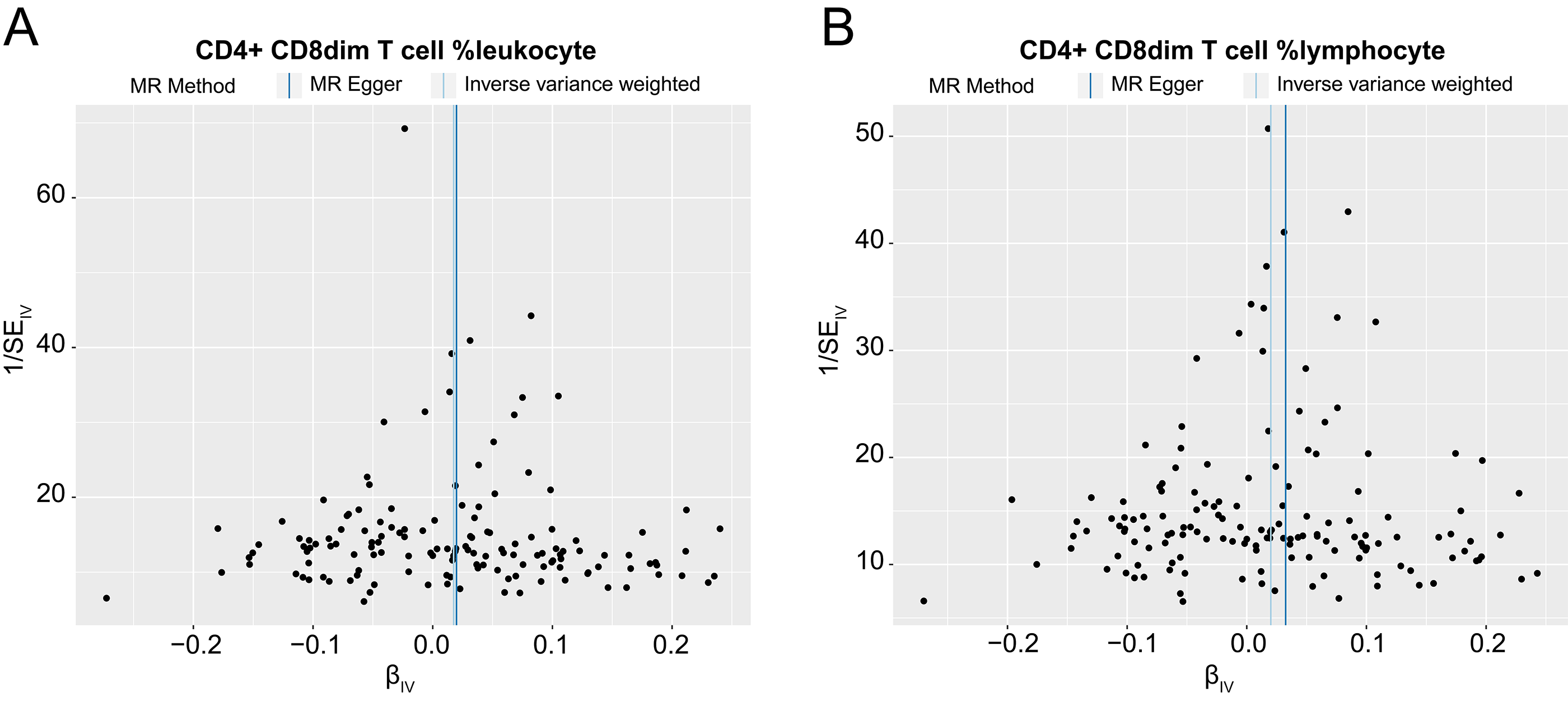

Supplement: Supplementary Figure S9 — Funnel plot for assessing publication bias in MR analysis specific to the GCST90027158 dataset, with immunophenotypic traits as exposures and AD as the outcome. [file Image_9.TIF]

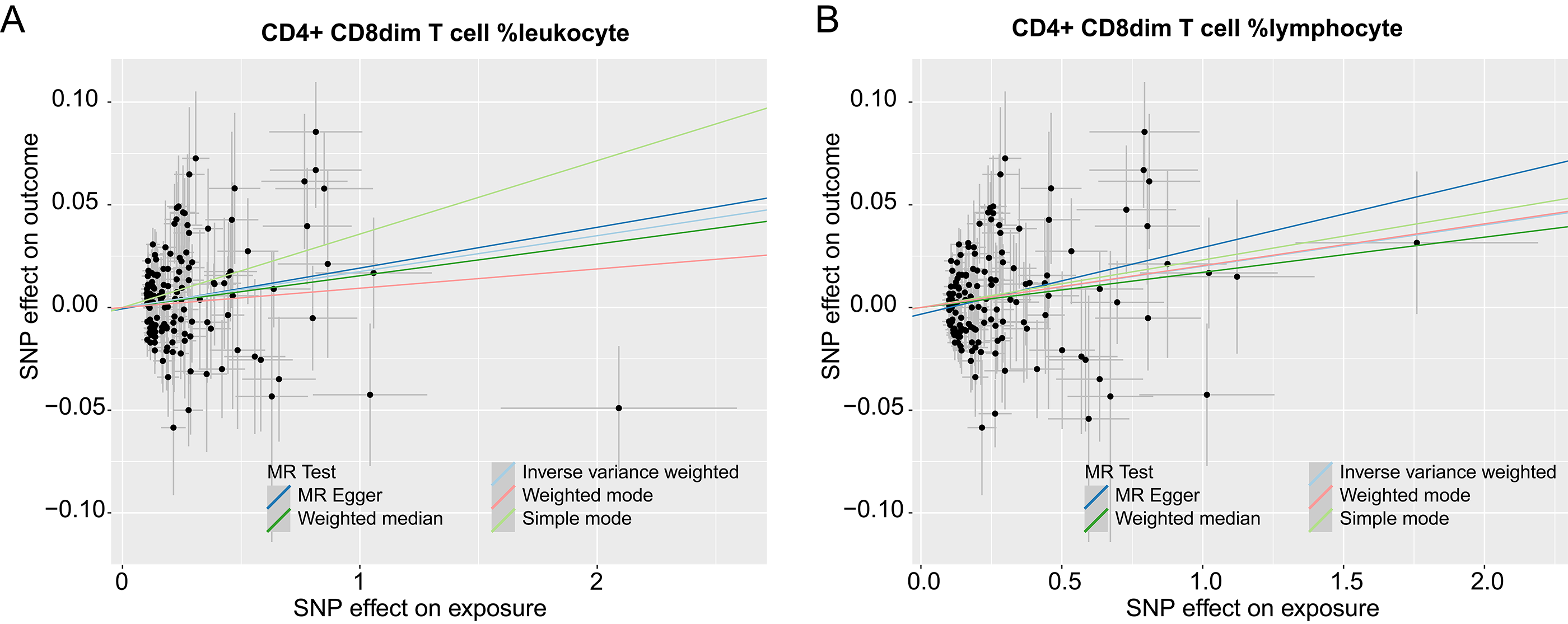

Supplement: Supplementary Figure S10 — Scatter plot for GCST90027158 dataset, displaying MR effect sizes for immunological exposures on AD outcome. [file Image_10.TIF]

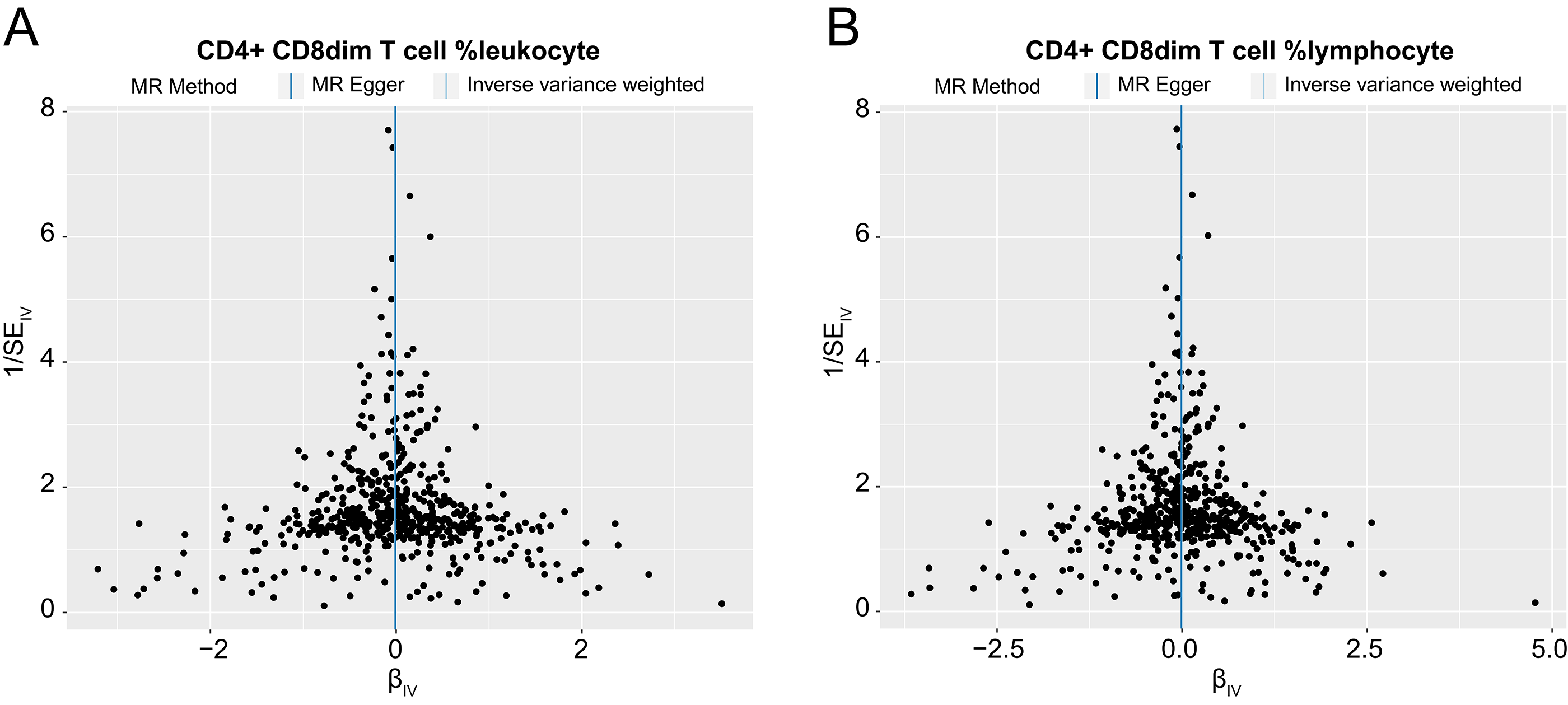

Supplement: Supplementary Figure S11 — Funnel plot for assessing publication bias in MR analysis of the GCST90027158 dataset, with AD as the exposure and immunophenotypic traits as outcomes. [file Image_11.TIF]

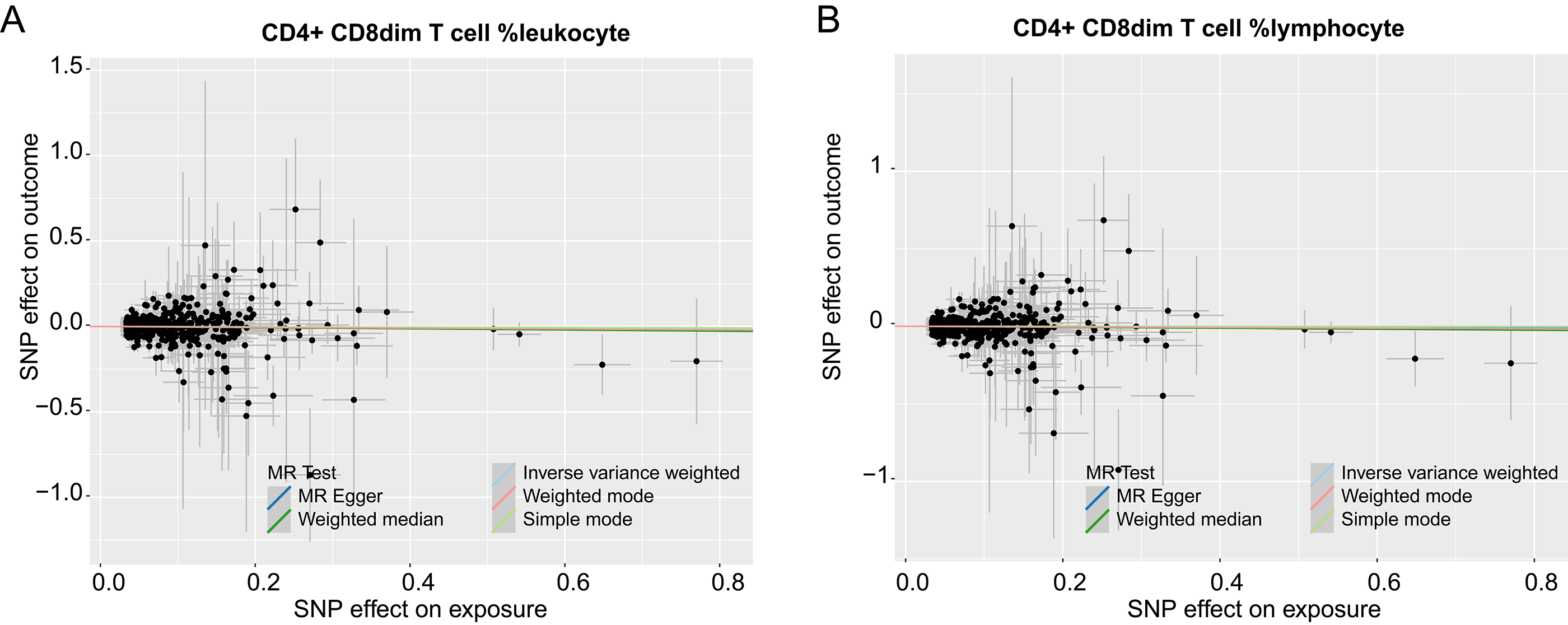

Supplement: Supplementary Figure S12 — Scatter plot for GCST90027158 dataset, presenting MR effect sizes for AD exposure on immunological outcomes. [file Image_12.TIF]
